# The Subcutaneous Implantable Cardioverter-defibrillator: New Features and Implant Techniques and Future Developments

**DOI:** 10.19102/icrm.2018.091104

**Published:** 2018-11-15

**Authors:** Nabil S. Zeineh, Jordan M. Prutkin

**Affiliations:** ^1^Electophysiology Section, Department of Cardiology, University of Washington, Seattle, WA, USA

**Keywords:** Hypertrophic cardiomyopathy, implantable defibrillator, subcutaneous ICD

## Abstract

The use of subcutaneous implantable cardioverter-defibrillators (S-ICDs) has increased over time. Device-based algorithms have been developed to reduce inappropriate shocks. New implant techniques have been developed including the two-incision technique and the placement of the generator submuscularly. More patients are being implanted without general anesthesia. This review summarizes the newest S-ICD features, surgical implantation methods, and device-related safety and efficacy findings.

## Introduction

Traditional implantable cardioverter-defibrillators (ICDs) consist of a pulse generator placed in the pectoral area and endocardial leads with high-voltage shocking coils. This necessitates the implantation of permanent intravascular hardware. Some of the periprocedural complications related to vascular access and intracardiac lead placement include pneumothorax, cardiac perforation, and lead dislodgement, while long-term complications such as device infection and lead malfunction due to exposure to perpetual cardiac motion as well stress from upper extremity bony and soft tissue compression can also occur.^1^ These complications are more common in younger, active patients. Of particular concern, device-related endocarditis carries significant mortality (up to 40%), with complete system extraction generally required for cure. Leads that are present for more than one year usually require laser- or mechanically-assisted extraction. The subcutaneous ICD (S-ICD) was designed to provide effective defibrillation while avoiding the complications related to the presence of hardware in the intravascular space. This device represents a novel development in the field of device therapy, and operator experience with the use of this technology has accrued significantly since its inception and approval. This review summarizes the newest S-ICD features, surgical implantation methods, and device-related safety and efficacy findings.

## Subcutaneous implantable cardioverter-defibrillator development

The first S-ICD, the SQRX from Cameron Health (San Clemente, CA, USA), was approved by the United States Food and Drug Administration (US FDA) in 2012. It was 15.7 mm thick, weighed 145 g, and was 69 mL in volume. Following the acquisition of Cameron Health by Boston Scientific (Natick, MA, USA), the second-generation EMBLEM™ device became available in 2015. This device was 12.7 mm thick, weighed 130 g, was 59.5 mL in volume, and included remote monitoring capabilities. In addition, software changes included the SMR8 algorithm, which, in a computer model, decreased T-wave oversensing (TWOS) by 40% with no change in the sensitivity of ventricular arrhythmia events.^2^ Then, in 2016, the current third-generation EMBLEM™ MRI S-ICD (Boston Scientific, Natick, MA, USA) was released. It is the same size as the second-generation device with a 7.3-year projected longevity and includes magnetic-resonance-imaging-conditional scanning and software changes, as described below. Separately, a new version of the S-ICD lead has also been released in which the suture sleeve is integrated directly on the lead, allowing the implanter to save time by alleviating the need to suture this down. This is in contrast to the old version of the device, in which the suture sleeve had to be manually sewn onto the lead.

A new feature of the current EMBLEM™ S-ICD device (Boston Scientific, Natick, MA, USA) is the SMART Pass arrhythmia discrimination algorithm, which is designed to reduce inappropriate shocks due to TWOS.^3^ It also includes an additional high-pass filter to reduce the amplitude of lower-frequency signals such as T-waves while maintaining sensing of higher-frequency R-waves. Limited data suggest that this algorithm reduces inappropriate shocks by 82% in comparison with the first-generation device and 71% in comparison with the second-generation device, respectively, with no change in time to detection or ventricular arrhythmia detection accuracy.^3^

The AF Monitor, another algorithm, stores episodes of suspected atrial fibrillation using ventricular scatter analysis, a measure of heart rate variability. To diagnose atrial fibrillation, the criterion of more than 80% of beats in a 192-beat window must be met, which may lead to the undercounting of shorter-duration episodes. In addition, a relatively regular ventricular response while in atrial fibrillation may be missed by the device. At least six minutes of atrial fibrillation must be present for the event to be stored, but the six minutes do not need to be continuous. One episode per day is stored for retrieval, with a total of up to seven episodes lasting 44 seconds in duration being kept. The device will report the percentage of the time spent in atrial fibrillation and the number of days where atrial fibrillation was detected within the last 90 days. In abstract form, this algorithm showed 100% specificity and 94% sensitivity for atrial fibrillation.^4^

## Subcutaneous implantable cardioverter-defibrillator screening

Screening continues to be important to reduce the risk of inappropriate shock. For this, electrodes are placed on the skin to approximate S-ICD sensing. The left leg electrode is placed in the fifth intercostal space on the midaxillary line, the left arm electrode is placed 1 cm lateral to the left side of the xiphoid midline, and the right arm electrode is placed 14 cm above the left arm electrode. If right-sided lead placement is to be completed, screening should be performed with the left and right arm electrodes located to the right of the sternum. The grounding electrode should be placed on the right lower abdomen.

Traditionally, screening is completed manually and can still be performed in this manner. The sweep speed is set at 25 mm/s with a gain of 5 mm/mV to 20 mm/mV, with a preference for the largest gain as long as there is no clipping of the QRS complex. The electrocardiogram (ECG) should be recorded for 10 seconds to 20 seconds with the patient both supine and standing, although sitting, left or right decubitus, or a prone position may be considered in circumstances in which there is concern of significant QRS or T-wave variability with body positioning. A patient screening tool made of a transparent plastic with colored profiles is used to assess the QRS complex and T-wave. The QRS is placed within the first part of the profile, with the specific profile chosen based on the size of the QRS complex. A screening pass is defined when all QRS complexes and T-waves fall within a profile, excepting occasional failures for premature ventricular complexes or paced beats (if a pacemaker is present). The same ECG lead should pass in the supine and standing positions. In general, the ZOOM™ Programmer (Boston Scientific, Natick, MA, USA) represents a good tool for screening. A 12-lead ECG machine can also be used, but, if borderline screening failure occurs due to small changes in filtering between machines, repeat screening with the ZOOM™ Programmer (Boston Scientific, Natick, MA, USA) may produce a positive result.^5^

There is now an automated screening tool (AST) built into the ZOOM™ Programmer (Boston Scientific, Natick, MA, USA). It uses the 3-Hz to 40-Hz band-pass filter in the programmer to match the sensing algorithm of the S-ICD. The AST needs at least six beats over 15 seconds to check the mean and standard deviation of the R-wave amplitude. If an R-wave is greater than one standard deviation in size from the mean, it is discarded and the mean is remeasured. This new average has to be greater than 0.47 mV or it is considered to be a screening failure. The second part of the AST is a measurement of the QRS:T-wave ratio; a screening pass is considered to demonstrate a result of more than 3.5:1.

The AST was designed to increase the speed of screening and reduce interobserver variability as a cause of screening failure. In addition, it more closely mimics the sensing filters of the S-ICD versus printing with manual evaluation.

## Subcutaneous implantable cardioverter-defibrillator implantation

**Figure 1:**
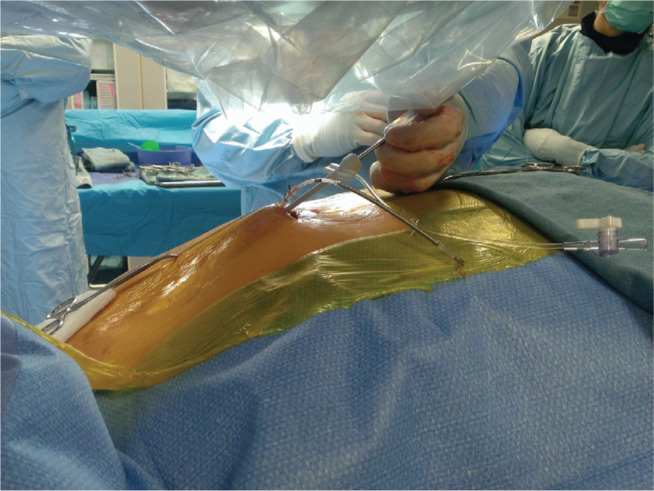
Insertion of the S-ICD lead with the two-incision technique. The tunneling tool has been inserted from the xiphoid incision toward the manubrium and the 11-French sheath is being advanced over the tunneling tool.

**Figure 2:**
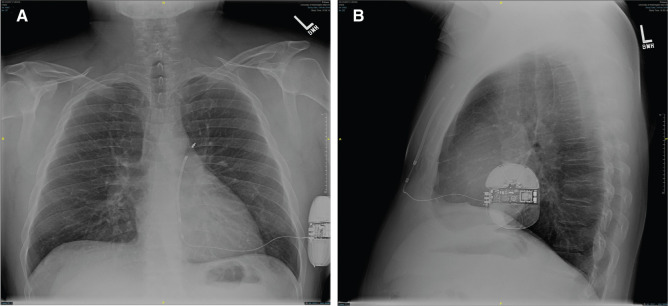
Postoperative posteroanterior **(A)** and lateral **(B)** chest X-rays demonstrating the position of the coil along the left parasternal border and the midaxillary position of the pulse generator.

Initially, most operators used the three-incision technique for placement of the S-ICD lead. However, over time, the two-incision technique has become the preferred technique, with more than half of the implants in the US using this.^6–8^ With this method, after the xiphoid incision is made, an 11-French or 12-French peel-away sheath is placed over the tunneling tool **(Figure 1)**. This tool is then advanced parasternally approximately 14 cm superiorly toward the manubrium. Next, the tunneling tool is removed, leaving the splittable sheath behind in the subcutaneous tissue, and the lead is inserted through the sheath, which can then be removed. The benefit of this technique is that it reduces procedure duration with equivalent safety and successful placement rates **(Figure 2)**.^7^

**Figure 3:**
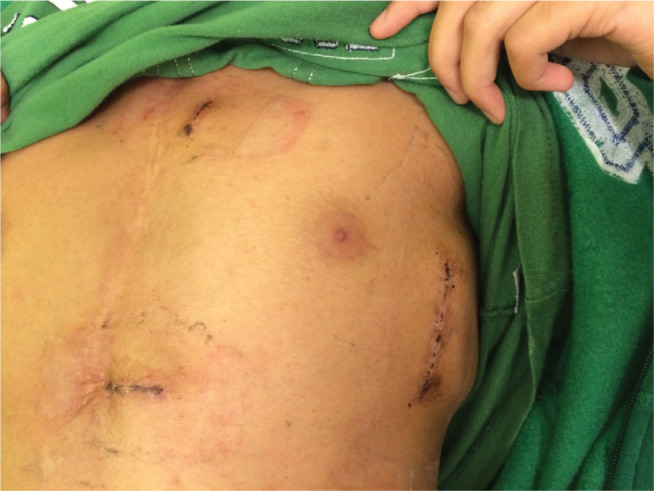
Submuscular S-ICD implanted using the three-incision technique between the serratus anterior and latissimus dorsi muscles of a 14-year-old, 40.8 kg male with complex congenital heart disease. The image shows the patient at 10 days after surgery.

Submuscular generator placement is an option to prevent erosion or for a more cosmetic appearance. One method is to position the generator between the serratus anterior and latissimus dorsi muscles **(Figure 3)**.^9–11^ This leads the device to be located more posteriorly than the standard subcutaneous generator location but cushions it to limit erosion.

A more complicated approach involves placing the device underneath the serratus anterior muscle.^12^ With this technique, the muscle slips of the serratus anterior are separated and a submuscular pocket is created beneath it. After the generator is placed in the pocket, the muscle slips are sutured together to keep the device in place.^7^ The generator can also be placed underneath the relatively thick fascia of the serratus anterior without going underneath the muscle. Care must be taken to prevent damage to the long thoracic nerve with these approaches.

There do not appear to be differences in outcomes between generator placement locations, but the use of the submuscular position is more painful for patients and the available data are limited with respect to long-term follow-up of the submuscular and subfascial generator locations.^7^ Overall, the depth of generator placement can be decided based on physician experience and patient factors such as body habitus to enhance patient acceptance of the device.

## Anesthesia and analgesia management

Most of the initial implant S-ICD procedures were performed under general anesthesia. Creation of the pocket and tunneling of the lead can be painful for patients, and most patients undergo defibrillation threshold testing (DFT) at implant. General anesthesia requires an anesthesiologist but allows the operator to focus solely on the procedure. However, general anesthesia and postanesthesia nursing care may not be widely available, and general anesthesia may lead to more hypotension, longer in-room time, and increased cost.^13^ In the S-ICD System Postapproval Study conducted by Boston Scientific (Natick, MA, USA), 64% of patients had general anesthesia.^8^ Several other options for anesthesia have been described in recent years.

Monitored anesthesia care (MAC) may be used in some cases, frequently with propofol.^14^ Mild to moderate sedation may be employed during some parts of the procedure, but, for tunneling and DFT, deeper sedation is often required. Local anesthesia should also be used to reduce sedation needs. Care should be taken to avoid oversedation and respiratory decompensation. The operative team should be familiar with airway maneuvers and an anesthesia team should be available if complications occur.

Nonanesthesiologist-administered sedation and analgesia (NASA) does not involve an anesthesiologist but involves sedation delivered by or under the guidance of the implanting physician. It may involve various agents including benzodiazepines, opioids, propofol, methohexital, etomidate, and/or nalbuphine.^15^ However, similar to using MAC, concerns about respiratory compromise or hypotension also exist, and NASA requires comparable cardiovascular and respiratory monitoring and a nurse dedicated solely to this task. Although this option is appealing for many hospitals, as it does not require an anesthesiologist and is less costly, there can be safety concerns. Staff must be appropriately trained to handle any potential complications, and the patient must be deemed eligible as a candidate to undergo NASA.

Recent reports have described regional nerve blocks used for breast surgery as an alternative method for S-ICD implantation anesthesia.^16–18^ These techniques target the thoracic intercostal nerves to provide a 13-hour to 14-hour analgesic effect. A recent report of 19 patients undergoing S-ICD implantation with two different nerve block techniques suggested it is feasible, safe, and can be performed in about 10 minutes.^18^

The serratus anterior plane block provides analgesia to the area in which the generator pocket is made by targeting the lateral cutaneous branches. Under ultrasound guidance, the T4–T5 level at the midaxillary line is imaged to find the plane between the latissimus dorsi and serratus anterior muscles.^18^ After confirming that this location is correct, 20 mL of 0.25% bupivacaine is injected.

The transversus thoracic muscle plane block provides analgesia to the sternum where the xiphoid incision and defibrillator coil portion of the lead are located by targeting the anterior branches. The plane between the transversus thoracis and internal intercostal muscles can be found near the left parasternal border at the T3–T4 level, and bupivacaine is injected here.^18^ MAC, NASA, or general anesthesia is still needed, but it is possible that regional nerve blocks can be used to limit postoperative pain.

## Efficacy and safety of the subcutaneous implantable cardioverter-defibrillator

The S-ICD underwent more than 10 years of preclinical and early clinical studies. Initial work demonstrated that the implantation position with the lowest defibrillation threshold was that of a left lateral pulse generator and an 8-cm shocking coil tunneled lateral to the left sternal border, which has subsequently become the currently used configuration.^19^

The device received Conformité Européenne approval in 2009. The trial that led to US FDA approval of the device in 2012 was an Investigational Device Exemption (IDE) study.^20^ This was a nonrandomized cohort study of 321 patients with ICD indications with an 11-month median follow-up period. The primary efficacy endpoint, acute conversion rate of induced ventricular fibrillation, was 94.7% with a single shock. Freedom from serious device-related complications at 180 days was 99% and the all-cause complication-free rate was 92.4%. The US FDA mandated a postapproval registry, and initial results of this observational study were published in 2017.^21^ The perioperative outcomes of 1,637 relatively sick patients from the US showed a similarly high, 30-day freedom from device- or procedure-related complications rate of 96.2%. Successful DFT, when attempted, occurred in 98.7% of patients.

A multinational registry of S-ICD recipients, mostly from European countries, called the Evaluation of Factors Impacting Clinical Outcome and Cost-effectiveness of the S-ICD (EFFORTLESS) study, has to date reported its early and midterm results.^22,23^ The latest report from this large registry was published in 2017.^24^ It included 985 patients, with a median follow-up of 3.1 years. The implantation indication in this cohort was primary prevention in 65%. The overall rate of inappropriate shocks was 11.7% and appeared to be lowered significantly by dual therapy zone programming. Most inappropriate shocks were due to TWOS. It is worth noting that only a minority (7.8%) of patients followed up with in this cohort had been implanted with the second-generation S-ICD, which has algorithms designed to reduce the rate of inappropriate shock by 30% to 40%.^2^

DFT is currently recommended for all patients undergoing S-ICD placement.^25^ In the S-ICD System Postapproval Study, there was a 98.7% conversion success rate.^8^ However, 1.6% of patients were noninducible and 12.3% did not undergo testing. In the EFFORTLESS cohort, 99.7% had a successful shock during testing, but some did require repositioning of the lead or generator to be successful, whereas 11.1% did not undergo defibrillating testing.^22^

Recent data from the ICD Registry from September 28, 2012 to April 1, 2016 demonstrated that only 70.7% of patients undergoing S-ICD implant in the US have undergone DFT testing.^26^ The greatest determinant of whether testing was performed was the implanting facility. Almost 7% of patients had an inappropriate safety margin, defined as the lowest energy successfully converting out of ventricular fibrillation of > 65 J. Importantly, DFT testing was not associated with in-hospital complications. Two smaller studies have suggested the safety of S-ICD implantation without DFT testing with reasonable patient outcomes and good shock efficacy.^27,28^

Overall, these findings show that, while recommended, not every patient can be a suitable candidate for DFT testing. For instance, the patient may be in atrial fibrillation and has not received appropriate anticoagulation, and there is concern that the shock will convert the rhythm to normal sinus. Some patients may also be thought to have too severe a degree of cardiac disease to undergo DFT. Larger studies are needed, however, before recommending that DFT testing is not needed for the S-ICD population.

One major concern with S-ICD therapy is regarding the lack of antitachycardia pacing (ATP) capability to treat monomorphic ventricular tachycardia (MMVT). Only 2.2% of patients in the EFFORTLESS cohort experienced more than one shock for MMVT during the 3.1 years of follow-up, a rate similar to the rate of 1.5% per year reported in the Sudden Cardiac Death in Heart Failure Trial (SCD-HeFT).^29^ The lack of ATP capability may not need to be a major deciding factor regarding what type of ICD should be offered. However, the lowest programmable treatment zone in the S-ICD is 170 bpm. Therefore, a history of slow ventricular tachycardia, especially if not controlled with antiarrhythmic therapy or ablation, would be a contraindication for S-ICD implant at present.

## The subcutaneous implantable cardioverter-defibrillator in hypertrophic cardiomyopathy

**Figure 4:**
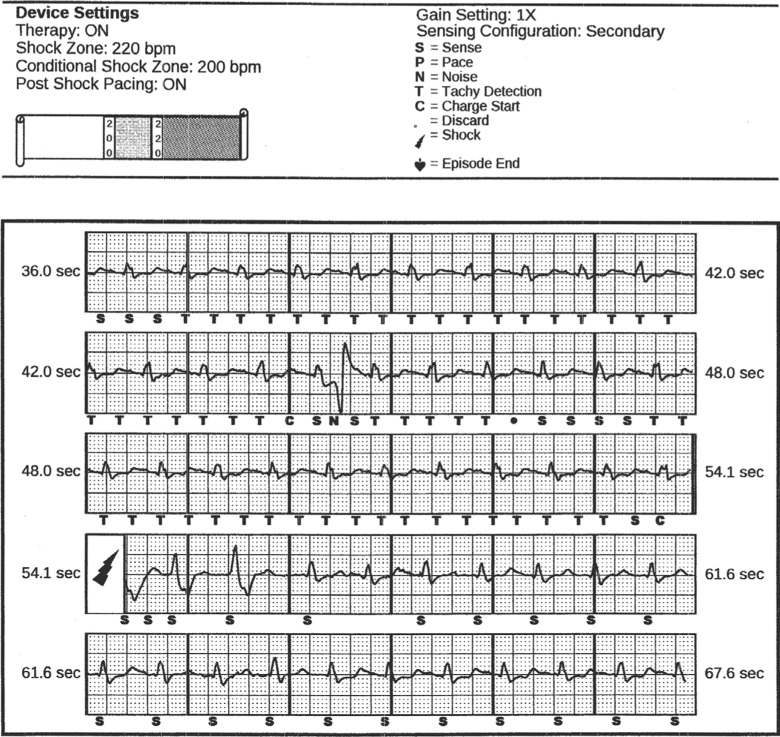
Inappropriate S-ICD shock due to T-wave oversensing in a patient with HCM. While the sinus rate is about 110 bpm, on the third row, double-counting of the T-wave has occurred (note the T marker, for tachycardia detection). The lightning bolt delineates the shock.

Patients with hypertrophic cardiomyopathy (HCM) are at varying risk for sudden cardiac death, and the ICD has been the main prophylactic measure for sudden cardiac death.^30,31^ The S-ICD seems to be a particularly attractive option in this patient population, given the frequently young age at implantation, the anticipated three to four decades of ICD therapy and lead failures, and the general lack of pacing needs. As mentioned previously, TWOS is the major cause of inappropriate shock delivery seen with the S-ICD system **(Figure 4)**, with increased QRS duration representing a risk factor for this untoward outcome. Patients with HCM can often have striking QRS and T-wave abnormalities. In a study of 27 patients with HCM who were screened for S-ICD candidacy, 85% passed the screening test and 59% proceeded with device implantation.^32^ DFT testing was performed in 15 patients, and a 65-J shock successfully restored sinus rhythm in all patients. No appropriate shocks were delivered after a median follow-up period of 17.5 months, but one patient received one inappropriate shock. Another study of 41 HCM patients who had ventricular fibrillation induced during DFT testing showed a success rate of 98.5%.^33^

Similarly, a cohort of patients with HCM was reported from the pooled IDE and EFFORTLESS registries.^34^ There were 99 patients with and 773 patients without HCM who received S-ICDs between 2009 and 2013 and who were followed up with for a median of 638 days. DFT testing was performed, with a rate of 95.5% successful defibrillation of induced ventricular fibrillation at ≤ 65 J. Overall ventricular arrhythmias were infrequent during the follow-up period (three cases of MMVT in three patients, all terminated with one shock). Interestingly, the rate of inappropriate shocks in patients with HCM was similar to that in patients without HCM (12.5% versus 10.7%; p = not significant). TWOS was the most common cause for inappropriate therapy in patients with HCM. In early studies, patients were programmed to a single shock zone, and it was recognized that dual-zone programming significantly reduced the rate of inappropriate shocks. In comparison, results from a large meta-analysis that included 2,190 patients with HCM showed that the rate of inappropriate shock using transvenous devices was on the order of 5%.^35^ Given the accumulating experience with S-ICD implantation as well as improved device programming and sensing algorithms, the rate of inappropriate shocks is expected to decrease. Judicious screening of HCM patients will also help to minimize the risk of inappropriate therapies. It has been reported that 7% to 16% of HCM patients fail screening in all three sensing vectors.^36^ This rate may be up to 38% in children with HCM.^37^ Although exercise ECG screening may not be necessary in the majority of S-ICD implants,^38^ patients with HCM may benefit from this additional screening step given the dynamic changes that may occur in the R- and T-wave morphologies in this patient population. These studies illustrate that a significant number of patients with HCM are potentially eligible for S-ICD implantation and showed device efficacy in terminating induced ventricular fibrillation.

## Future directions

With the introduction of the S-ICD came the beginning of the elimination of the “weakest link” in the ICD system, the transvenous lead.^39^ The S-ICD continues to have shortcomings, however, one of which is that it does not offer bradycardia pacing or ATP. Futures versions of the device could potentially be paired with a leadless pacemaker, with communication occurring between the devices. This has been demonstrated in an animal model wherein the S-ICD sensed the ventricular arrhythmia and commanded the leadless pacemaker to deliver ATP.^40^ In 99% of episodes, there was successful communication between the S-ICD and leadless pacemaker. It is possible that future versions of this combination system will include sensing from the leadless pacemaker to enhance discriminatory ability and reduce inappropriate shocks from TWOS.

Some randomized controlled trials evaluating the efficacy of the S-ICD will shed light on important clinical issues. The Multicenter Automatic Defibrillator Implantation Trial with S-ICD (MADIT S-ICD) study is currently ongoing (NCT02787785).^41^ Patients with diabetes mellitus and ischemic cardiomyopathy have a higher rate of sudden cardiac death than do those without diabetes mellitus, even when the ejection fraction is more than 35%.^42^ MADIT S-ICD was expected to randomize 1,800 patients with diabetes mellitus, prior myocardial infarction, ejection fraction of 36% to 50%, and age ≥ 65 years to an S-ICD or usual medical therapy arm with a primary endpoint of all-cause mortality. In addition, the Prospective, Randomized Comparison of Subcutaneous and Transvenous ICD Therapy (PRAETORIAN) study is an ongoing clinical trial (NCT01296022) randomizing patients with a class I or IIa indication for an ICD without pacing indications to either a transvenous or subcutaneous device.^43^ The primary endpoint is ICD-related adverse events.

## Conclusion

The S-ICD is no longer an emerging therapy.^44^ At this point, enough data support its use in many different populations. Surgical and anesthetic/analgesic techniques are evolving to improve speed of implant, enhance safety, and reduce postoperative pain. Future iterations will continue to enhance arrhythmia discrimination, possibly with the pairing of a leadless pacemaker.
